# Expression of c-fos Was Associated with Clinicopathologic Characteristics and Prognosis in Pancreatic Cancer

**DOI:** 10.1371/journal.pone.0120332

**Published:** 2015-03-19

**Authors:** Jun-Chao Guo, Jian Li, Yu-Pei Zhao, Li Zhou, Quan-Cai Cui, Wei-Xun Zhou, Tai-Ping Zhang, Lei You

**Affiliations:** 1 Department of General Surgery, Peking Union Medical College Hospital, Chinese Academy of Medical Sciences/Peking Union Medical College, Beijing, China; 2 Department of Pathology, Peking Union Medical College Hospital, Chinese Academy of Medical Sciences/Peking Union Medical College, Beijing, China; University of North Carolina School of Medicine, UNITED STATES

## Abstract

It has long been regarded that pancreatic cancer (PC) is a life-threatening malignant tumor. Thus, much attention has been paid for factors, especially relative molecules, predictive for prognosis of PC. However, c-fos expression in PC was less investigated. In addition, its association with clinicopathologic variables and prognosis remains unknown. In the present study, expression of c-fos was detected by tissue microarray-based immunohistochemical staining in cancer and adjacent tissues from 333 patients with PC. The staining results were correlated with clinicopathologic parameters and overall survival. Furthermore, prognostic significance of c-fos in subsets of PC was also evaluated. It was shown that low expression of c-fos was more often in cancer than in adjacent tissues of PC (*P*<0.001). Besides, high cancerous c-fos expression was significantly associated with tumor site and T stage, whereas peri-neural invasion was of a borderline significant relevance. Log-rank test revealed that high expression of c-fos in cancer tissues was a significant marker of poor overall survival, accompanied by some conventional clinicopathologic variables, such as sex, grade, peri-neural invasion, T and N stages. More importantly, cancerous c-fos expression was identified as an independent prognosticator in multivariate analysis. Finally, the prognostic implication of c-fos expression was proven in four subsets of patients with PC. These data suggested that c-fos expression was of relationships with progression and dismal prognosis of PC.

## Introduction

Pancreatic cancer (PC) is one of most life-threatening solid neoplasms, because this type of malignancy carries the mortality almost equal to its incidence [1]. It was recently summarized that improvement in long-term prognosis of PC, although surgical resection was more and more applied, was not achieved within two decades [2]. Therefore, exploration of prognostic markers of PC has been the hotspot of study. Thus far, many clinical and pathologic ones, such as lymph node status, neural invasion, tumor marker levels and resection margin, were identified [3–8]. On the other hand, impacts of molecules involved in tumor pathogenesis and progression on prognosis of PC were gradually valued and recently reviewed [9,10]. Of course, more molecular prognosticators need to be discovered.

It has been well known that c-fos (the human homolog of the retroviral oncogene v-Fos), one of major components of activator protein (AP)-1 complex, mediates cancer growth, angiogenesis, invasion and metastasis, by regulating expressions of down-stream genes [11]. The findings that c-fos expression was significantly associated with recurrence, metastasis and poor prognosis in squamous lung cancer, breast cancer and osteosarcoma provided its potential as a proto-oncogene [12–14]. However, other data suggested that patients with high c-fos expression carried favorable survival in colorectal, ovarian and gastric cancers [15–18]. Thus, c-fos expression and its biological effects might be tissue-type specific. In PC, quite different positive rates of c-fos and its adverse associations with malignant phenotypes, such as lymph node metastasis and multidrug resistance, were previously reported [19–22]. However, its clinicopathologic and prognostic significances in PC remain unknown.

In the present study, the authors aimed to address the issues, based on a large Chinese cohort with PC.

## Materials and Methods

### Patients

The study cohort consisted of 333 patients with PC, including 206 males and 127 females. Ages ranged from 34 to 85 (median: 60) years. Patient selection criteria: (1) Histologically proven adenocarcinoma; (2) Surgically resected specimens available; (3) Clinicopathologic data available. Table 1 summarizes the clinicopathologic characteristics of the patients. The paired formalin-fixed paraffin-embedded cancer and non-cancer tissues for patients were used. The written informed consent of all patients was obtained. The project (including its consent procedure) was approved by the Ethics Committee of Peking Union Medical College Hospital.

**Table 1:** The criteria on the grades integrating positive ratio and intensity.

The ratio of positive cells	Staining intensity	Final grades	Implication
0–10% (1)	Faint (1)	**1**	**Low**
	Moderate (2)	**2**	**Low**
	Intensive (3)	**3**	**Low**
>10–≥50% (2)	Faint (1)	**2**	**Low**
	Moderate (2)	**4**	**Low**
	Intensive (3)	**6**	**High**
>50% (3)	Faint (1)	**3**	**Low**
	Moderate (2)	**6**	**High**
	Intensive (3)	**9**	**High**

### Construction of Tissue Microarray (TMA)

Tissue microarray construction was performed according to methods and standards same with those previously reported [23].

### Immunohistochemical Staining

A rabbit anti-human polyclonal antibody for c-fos (sc-52, dilution: 1: 50, Santa Cruz Biotechnology, Inc., Santa Cruz, CA) and a two-step immunostaining kit (EnVision^TM^+kit, Dako, Denmark) were used for staining. The blocking peptide (Santa Cruz) was adopted for specificity validation of the primary antibody. Slides were stained in accordance with the method that was previously applied [23]. Non-immune rabbit serum at the same dilution was adopted as the negative control.

### Evaluation of Staining Results

Two independent pathologists who were blinded to the clinicopathologic and survival data (Q.C. C. and W.X. Z.) evaluated staining results. The brown nuclear and/or cytoplasm coloration was defined as the positive signal. According to previous suggested [24], the ratio of positive cells was divided into three grades (0–10%, grade 1; >10-≥50%, grade 2; >50%, grade 3). On the other hand, staining intensity was also scored to three groups (faint, score 1; moderate, score 2; intensive, score 3). The final grade was obtained by multiplication of positive ratio grade and intensity score (shown in Table 1), and separated into low (final grade <6) and high expressions (final grade ≥6).

### Follow-Up

Follow-up (median: 14 months; range: 2 to 87 months) was performed for 229 patients, mainly through outpatient clinic, telephone and mail. During the follow-up term, 170 patients have died.

### Statistical Analysis

McNemar's test and Pearson’s correlation analysis were used to compare c-fos expression between cancer and adjacent non-cancer tissues, and to show the correlation between them. The association between c-fos expression and clinicopathologic characteristics was revealed by Chi-square test. Kaplan-Meier method and log-rank test were applied to estimate overall survival and their differences. Multivariate Cox regression (Proportional hazard model) analysis was performed for identification of independent prognostic factors. All the analyses were finished by statistical software package SPSS11.5 (SPSS Inc, Chicago, IL). A *P* value less than 0.05 was defined as statistically significant.

## Results

### Expression of c-fos and Its Association with Clinicopathologic Characteristics in PC

**Fig 1:**
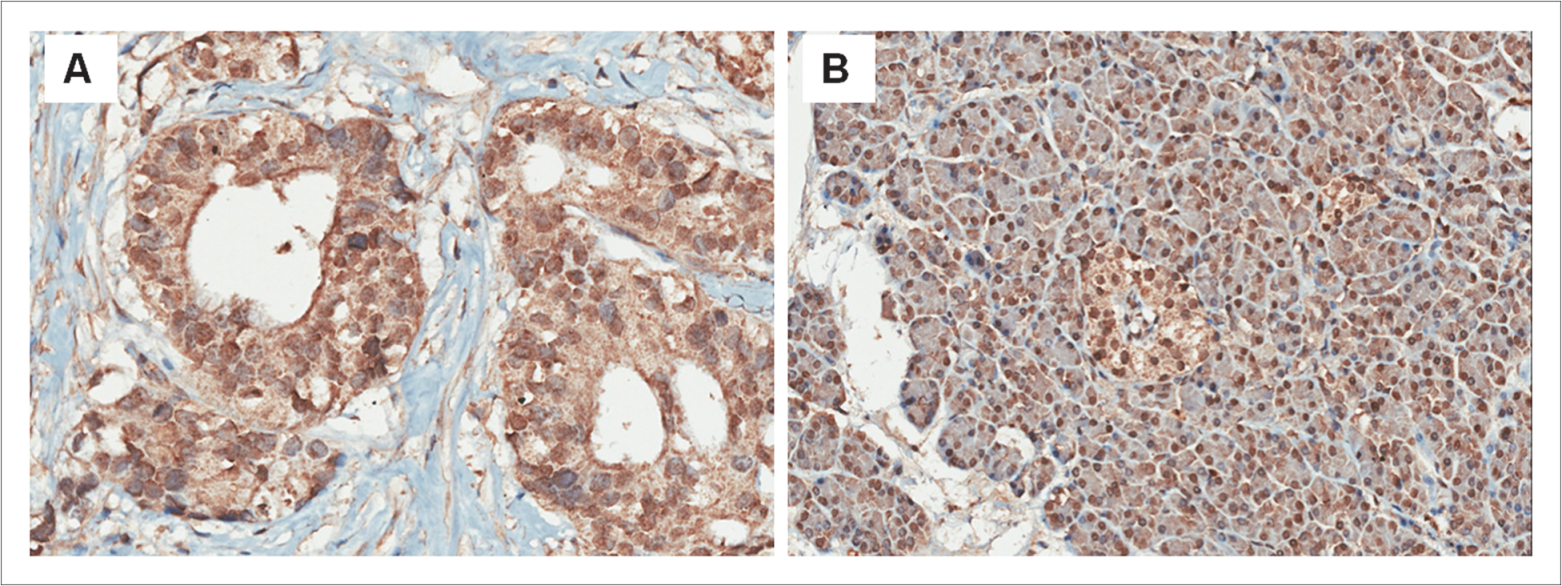
Expression of c-fos in pancreatic cancer. (A) High expression in cancer tissue (original magnification ×200). (B) High expression in adjacent non-cancer tissue (original magnification ×200).

It was shown that the positive signal mainly located in cell nucleus, and being also seen in cytoplasm (Fig. 1). After treatment with the blocking peptide, the finding that positive signal disappeared (S1 Fig.) validated the specificity of the primary antibody. Low and high expressions were found in cancer tissues from 167 and 166 patients with PC, and adjacent non-cancer tissues from 63 and 270 individuals. Low c-fos expression was more common in cancer than in adjacent tissues (*P*<0.001, McNemar's test). There were significant correlation between c-fos expression in cancer tissues and that in adjacent non-cancer tissues (*P*<0.001, Pearson’s correlation analysis). Chi-square analysis showed that high cancerous c-fos expression was significantly frequent in non-head and T3 tumors, compared with head and T1–2 ones, respectively (Table 2, *P* = 0.013 and 0.017). Moreover, c-fos expression in cancer tissues was of marginally significant relationship with perineural invasion (Table 2, *P* = 0.069). Expression of c-fos in adjacent tissues was not related to clinicopathologic parameters included (data not shown).

**Table 2:** Associations of cancerous c-fos expression with clinicopathologic characteristics of PC.

		c-fos expression in TT		
Variables	Number	High (%)	Low (%)	*P* *
**Sex**				0.331
Male	206	107 (51.9)	99 (48.1)	
Female	127	59 (46.5)	68 (53.5)	
Age				0.847
≥65 years	112	55 (49.1)	57 (50.9)	
<65 years	221	111 (50.2)	110 (49.8)	
Tumor site				**0.013**
Head	202	90 (44.6)	112 (55.4)	
Non-head	115	68 (59.1)	47 (40.9)	
Tumor size				0.812
>4cm	124	60 (48.4)	64 (51.6)	
≤4cm	199	99 (49.7)	100 (50.3)	
Grade				0.350
G1-2	231	109 (47.2)	122 (52.8)	
G3-4	71	38 (53.5)	33 (46.5)	
PNI				0.069
Present	160	88 (55.0)	72 (45.0)	
Absent	150	67 (44.7)	83 (55.3)	
T stage				**0.017**
T1-2	222	100 (45.0)	122 (55.0)	
T3	101	60 (59.4)	41 (40.6)	
N stage				
N0	170	87 (51.2)	83 (48.8)	
N1	134	65 (48.5)	69 (51.5)	

PC, pancreatic cancer; TT, tumor tissue; G1, well differentiated; G2, moderately differentiated; G3, poorly differentiated; G4, undifferentiated; PNI, peri-neural invasion; T, tumor; N, lymph node.

* Chi-square test.

### Prognostic Significance of c-fos in the Whole Cohort of PC

**Fig 2:**
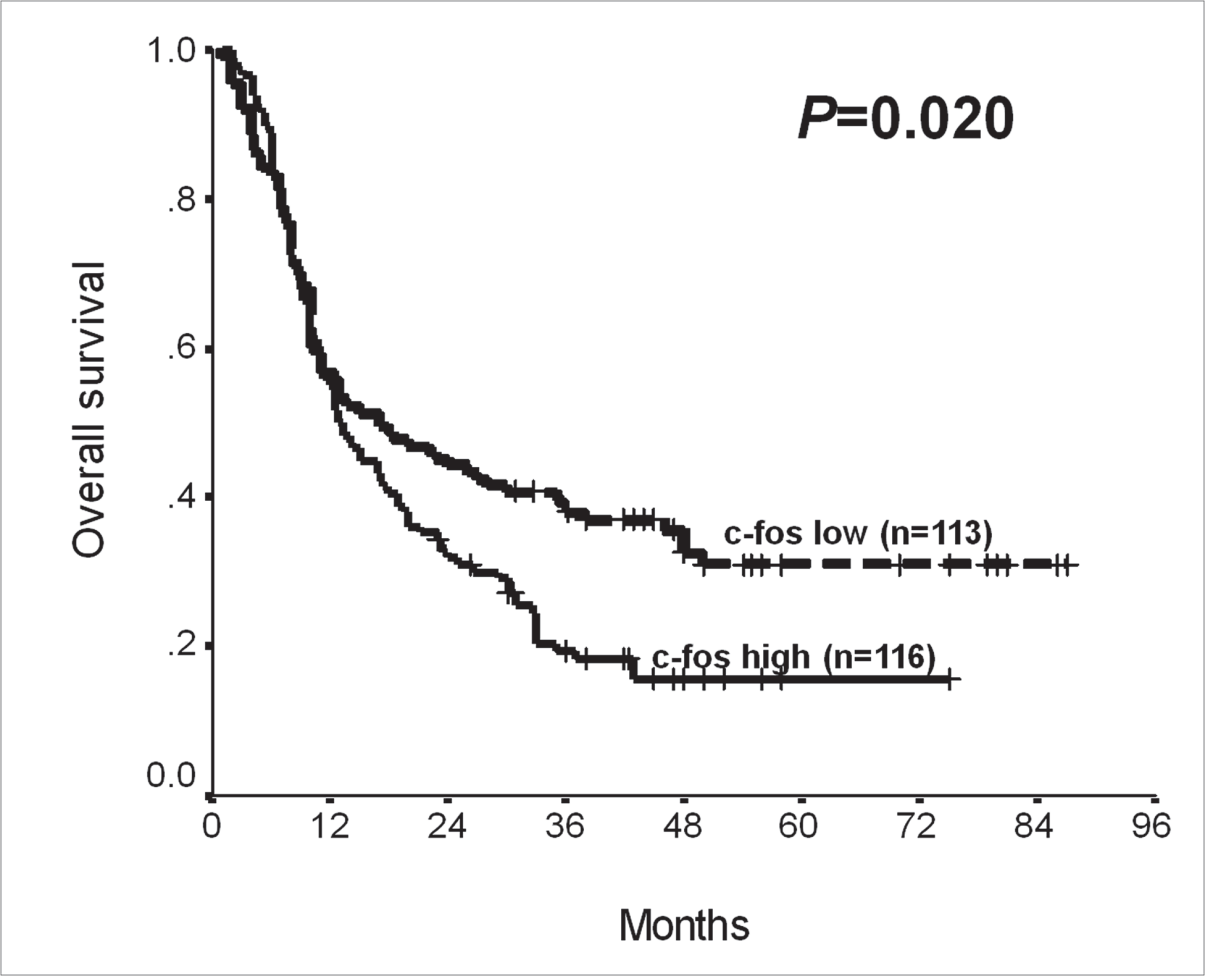
Post-surgical overall survival of pancreatic cancer according to c-fos expression in cancer tissues (Log-rank test; *P* = 0.020).

Univariate analysis in the whole cohort of PC showed that high cancerous c-fos expression predicted shortened overall survival (*P* = 0.020; Fig. 2 and Table 3), in addition to sex, histological grade, perineural invasion, T and N stages (*P*<0.05; Table 3). Multivariate Cox regression defined c-fos expression as one of independent prognostic indicators for overall survival of PC (*P* = 0.022; Table 3).

**Table 3:** Prognostic factors of PC after resection (the whole cohort).

		OS (Univariate)			OS (Multivariate)		
Variables	Number (*n*)	median±SE	95%CI	*P* ^*^	RR	95%CI	*P* ^#^
Sex				0.003			0.014
Male	148	13.00±1.45	10.15–15.85		1.597	1.098–2.324	
Female	81	19.60±11.26	0–41.68		1		
Age				0.941			
≥65 years	73	12.50±2.74	7.13–17.87				
<65 years	156	14.30±2.22	9.94–18.66				
Tumor site				0.464			
Head	134	14.30±2.85	8.71–19.89				
Non-head	87	12.80±2.64	7.62–17.98				
Tumor size				0.919			
>4cm	93	12.50±1.40	9.75–15.25				
≤4cm	133	18.00±2.85	11.41–22.59				
Grade				**0.004**			**<0.001**
G1-2	154	18.80±3.50	11.95–25.65		1		
G3-4	57	10.00±0.75	8.53–11.47		1.995	1.367–2.911	
PNI				**0.002**			**0.042**
Present	108	11.20±0.90	9.43–12.97		1.440	1.013–2.048	
Absent	110	21.30±4.26	12.94–29.66		1		
T stage				**0.028**			**0.021**
T1-2	152	17.00±3.15	10.82–23.18		1		
T3	75	12.50±1.73	9.11–15.89		1.549	1.070–2.242	
N stage				**<0.001**			**0.001**
N0	126	20.00±3.84	12.47–27.53		1		
N1	89	11.00±1.16	8.73–13.27		1.744	1.241–2.451	
c-fos expression				**0.020**			**0.022**
High	116	12.80±1.35	10.16–15.44		1.520	1.062–2.176	
Low	113	17.00±4.70	7.78–26.22		1		

PC, pancreatic cancer; SE, standard error; CI, confidence interval; RR, relative risk; G1, well differentiated; G2, moderately differentiated; G3, poorly differentiated; G4, undifferentiated; PNI, peri-neural invasion; T, tumor; N, lymph node.

* Log-rank test

^#^ Cox regression test.

### Influences of c-fos Expression on Overall Survival in Subsets of PC

**Fig 3:**
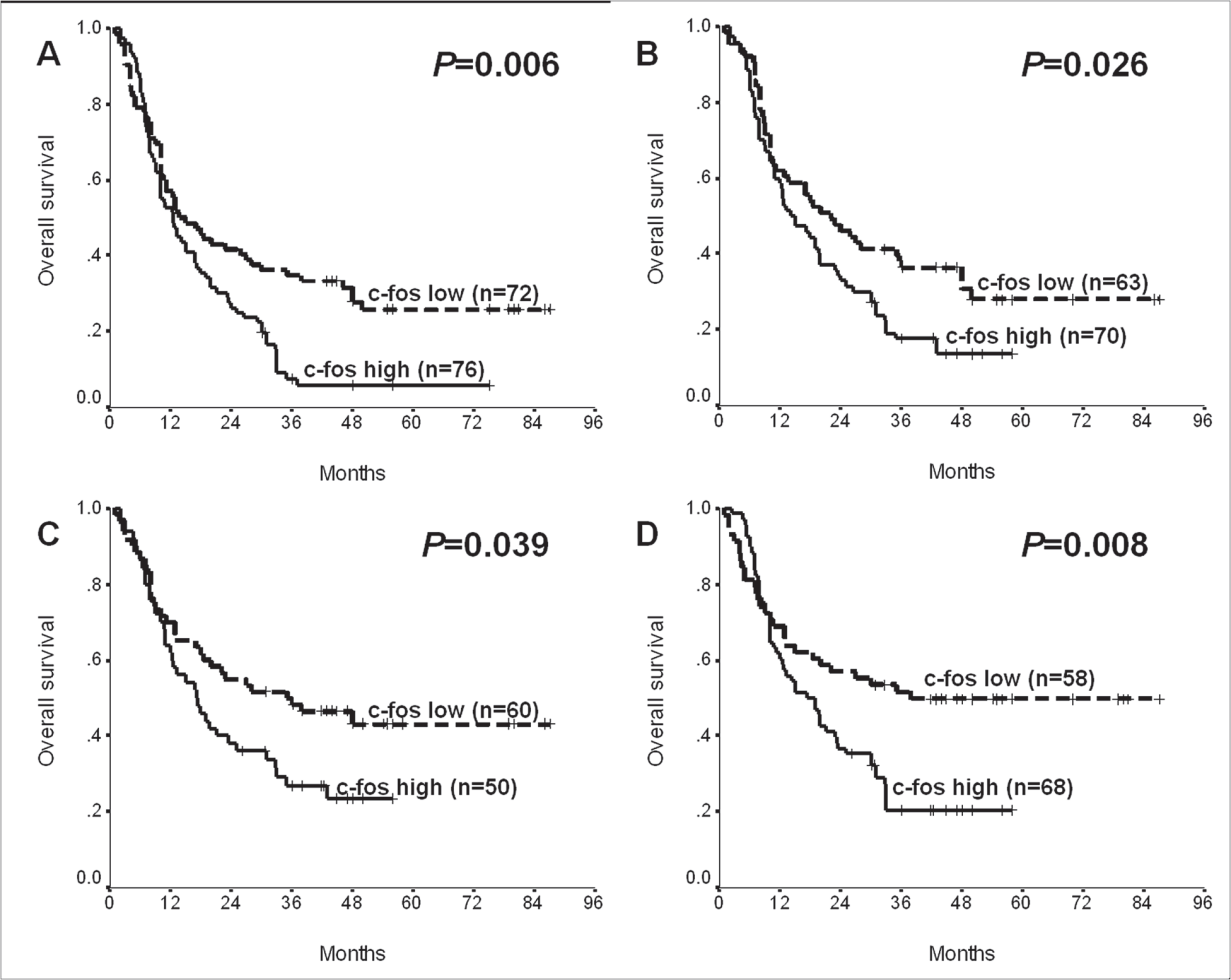
Prognostic significances of cancerous c-fos expression in subsets of PC after resection. (A) Male patients (*P* = 0.006). (B) Tumors ≤4cm (*P* = 0.026). (C) Tumors without peri-neural invasion (*P* = 0.039). (D) N0 tumors (*P* = 0.008). N, lymph node.

Because the present study evaluated eight clinicopathologic variables, patients were divided into sixteen subsets. Univariate analysis defined cancerous c-fos expression as a significant predictor for overall survival in four ones, i.e. male patients, tumors ≤4cm, N0 tumors and those without perineural invasion (*P*<0.05; Fig. 3). Cox regression analysis established that c-fos expression in cancer tissues was of independent prognostic implication in males, N0 tumors and those without perineural invasion (*P*<0.05; Table 4).

**Table 4:** Multivariate Cox regression analysis for prognostic significance of cancerous c-fos expression in some subsets of PC after resection.

Subsets	RR	95%CI	*P* ^#^
Males	1.722	1.147–2.586	**0.009**
≤4cm	1.470	0.969–2.230	0.070
Without PNI	1.746	1.036–2.943	**0.036**
N0	1.718	1.012–2.917	**0.045**

PC, pancreatic cancer; RR, relative risk; CI, confidence interval; PNI, peri-neural invasion; N, lymph node.

^#^ Cox regression test.

## Discussion

As an important member of the fos family of transcription factors, c-fos was demonstrated to be a key regulator of proliferation, differentiation, angiogenesis, invasion and metastasis of cancer, through AP-1-related mechanisms [11]. However, it is interesting that c-fos plays different, even opposite roles in different cancers. In breast and bladder cancers as well as glioma cells [25–28], c-fos seems to have pro-oncogenic properties, unlike in ovarian cancer [29]. Although in a same kind of malignancy, PC, results concerning expression and phenotypes of c-fos remain to be inconsistent. Using immunohistochemistry for a few individuals, Lee et al. [19] found that c-fos was highly expressed in PC tissues, but only weak expression was observed in a small number of patients enrolled in another study [20]. Furthermore, the findings that c-fos was down-regulated in PC with lymph node metastasis and increased c-fos expression induced multidrug resistance 1 (MDR1) in PC cells indicate the complex roles of the molecule in PC [21,22]. Other investigations that showed that c-fos contributes to cell motility [30] and functions as one of targets of some tumor suppressor genes [31,32], support its oncogenic potential in PC. In the current investigation, expression of c-fos was first detected for more than 300 patients with PC. It is unexpected that low c-fos expression is more frequent in cancer than in adjacent non-cancer tissues (McNemar's test). Then, the positive/potentially positive associations between high c-fos expression and later T stage as well as presence of perineural invasion were shown. The results indicate that c-fos might play crucial roles in both initiation and progression of PC. Previous work found that c-fos induced inflammation-mediated tumorigenesis [33]. In addition, chronic pancreatitis (CP) was well known as a significant etiological factor of PC [34]. Therefore, whether c-fos plays an important role in the process from CP to PC might be of interest. It was suggested that K-ras, a crucial mutated gene in both CP and PC, activated Raf/MEK/extracellular signal-regulated kinase (ERK) pathway through a complex cascade [34], whereas ERK, a kind of mitogen-activated protein kinase (MAPK), was involved in activation of c-fos [35]. Thus, high c-fos expression in adjacent non-cancer tissues in the current study might provide a preliminary clue of its impacts on conversion from inflammation to malignancy in the pancreas. On the other hand, cancerous c-fos expression was revealed to be significantly and marginally significantly associated with T stage and perineural invasion, indicating that this transcriptional factor might be a promoter of PC progression. Taken together, our data supported that c-fos serves as a proto-oncogene in the course from malignant transformation to progression in PC, different with previous one showing down-regulation of c-fos in PC with lymph node metastasis [21]. In the future, detailed explorations for phenotypes and relative mechanisms worth to be done.

It was shown that c-fos was linked to poor or good prognosis in many types of cancers [12–18]. However, its impact on outcome of PC has not been elucidated. Here, we report that high c-fos expression in cancer tissues was associated with unsatisfactory overall survival in both univariate and multivariate analyses, presenting its power as a strong predictor of gloomy prognosis in PC. The results that c-fos was of independent prognostic significance in some subsets of PC strengthen its prognostic role. These findings, supported by the positive relationship between c-fos expression and malignant phenotypes of PC established in the present study, are consistent with those derived from osteosarcoma, lung and breast cancers [12–14]. No doubt these survival analysis results also indicate the function of c-fos as a proto-oncogene.

In conclusion, our data demonstrated that c-fos expression is related to progression and dismal prognosis of PC. Thus, this protein might be a novel molecular maker of PC.

## Supporting Information

S1 Fig Specificity validation of the primary antibody against c-fos in pancreatic cancer. (A) With blocking peptide (original magnification ×200). (B) Without blocking peptide (original magnification ×200).
(TIF)
