# Supplementary figures and images for: Expression of c-fos Was Associated with Clinicopathologic Characteristics and Prognosis in Pancreatic Cancer
Source: PLoS One. 2015 Mar 19;10(3):e0120332. doi: 10.1371/journal.pone.0120332 (PMC4366380; doi:10.1371/journal.pone.0120332)

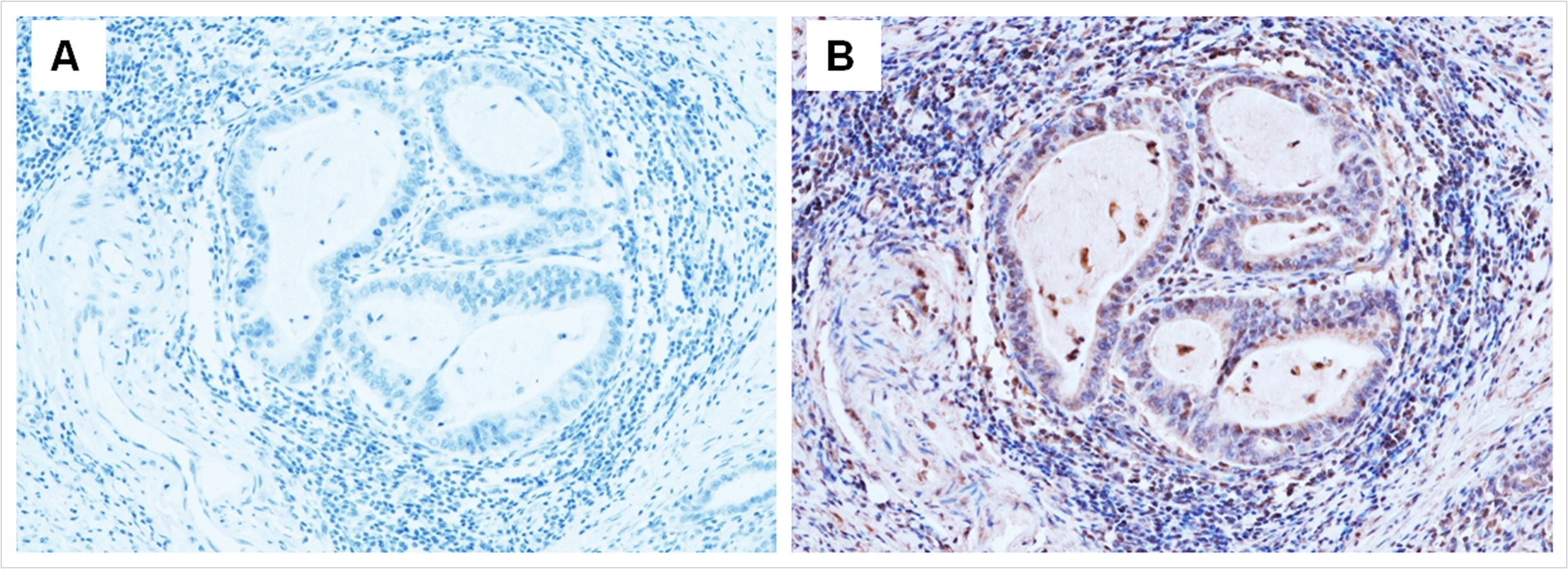

Supplement: S1 Fig — (A) With blocking peptide (original magnification ×200). (B) Without blocking peptide (original magnification ×200). (TIF) [file pone.0120332.s001.tif]
